# Clinicopathological factors and survival outcomes of signet-ring cell and mucinous carcinoma versus adenocarcinoma of the colon and rectum: a systematic review and meta-analysis

**DOI:** 10.1007/s12672-021-00398-6

**Published:** 2021-02-22

**Authors:** Michael G. Fadel, George Malietzis, Vasilis Constantinides, Gianluca Pellino, Paris Tekkis, Christos Kontovounisios

**Affiliations:** 1 Department of Colorectal Surgery, Chelsea and Westminster Hospital, London, UK; 2 Department of Surgery and Cancer, Imperial College, London, UK; 3 Department of Colorectal Surgery, Royal Marsden Hospital, London, UK; 4 Department of General Surgery, Evangelistria Medical Centre, Nicosia, Cyprus; 5 Department of Advanced Medical and Surgical Sciences, Università degli Studi della Campania ‘Luigi Vanvitelli’, Naples, Italy

**Keywords:** Colorectal cancer, Signet-ring cell cancer, Mucinous cancer, Survival, Recurrence

## Abstract

**Background:**

Histological subtypes of colorectal cancer may be associated with varied prognostic features. This systematic review and meta-analysis aimed to compare clinicopathological characteristics, recurrence and overall survival between colorectal signet-ring cell (SC) and mucinous carcinoma (MC) to conventional adenocarcinoma (AC).

**Methods:**

A literature search of MEDLINE, EMBASE, Ovid and Cochrane Library was performed for studies that reported data on clinicopathological and survival outcomes on SC and/or MC versus AC from January 1985 to May 2020. Meta-analysis was performed using random-effect models and between-study heterogeneity was assessed.

**Results:**

Thirty studies of 1,087,055 patients were included: 11,510 (1.06%) with SC, 110,179 (10.13%) with MC and 965,366 (88.81%) with AC. Patients with SC were younger than patients with AC (WMD − 0.47; 95% CI − 0.84 to –0.10; *I*^2^ 88.6%; p = 0.014) and more likely to have right-sided disease (OR 2.12; 95% CI 1.72–2.60; *I*^2^ 82.9%; p < 0.001). Locoregional recurrence at 5 years was more frequent in patients with SC (OR 2.81; 95% CI 1.40–5.65; *I*^2^ 0.0%; p = 0.004) and MC (OR 1.92; 95% CI 1.18–3.15; *I*^2^ 74.0%; p = 0.009). 5-year overall survival was significantly reduced when comparing SC and MC to AC (HR 2.54; 95% CI 1.98–3.27; *I*^2^ 99.1%; p < 0.001 and HR 1.38; 95% CI 1.19–1.61; *I*^2^ 98.6%; p < 0.001, respectively).

**Conclusion:**

SC and MC are associated with right-sided lesions, advanced stage at presentation, higher rates of recurrence and poorer overall survival. This has strong implications towards surgical and oncological management and surveillance of colorectal cancer.

**Supplementary Information:**

The online version contains supplementary material available at 10.1007/s12672-021-00398-6.

## Introduction

Colorectal cancer is the third most common diagnosed malignancy and the fourth leading cause of cancer death in the world [1–3]. There were 1.8 million new cases and approximately 861,000 deaths as a result of colorectal cancer in 2018 [4]. Recent data from the United States Surveillance, Epidemiology, and End Results (SEER) database and other Western cancer registries suggest that colorectal cancer is increasing in adults under the age of 50 years [5–8]. In the United States, the incidence of colorectal cancer in adults under the age of 50 years steadily increased at a rate of 2% per year from 1995 to 2016 [9]. The cause of this rise in colorectal cancer in younger adults has not yet been established.

The majority of colorectal cancers are adenocarcinomas with three major subtypes: conventional adenocarcinoma (AC), mucinous (MC) and signet-ring cell (SC) adenocarcinomas [10]. MC account for 5–15% of colorectal adenocarcinomas while SC accounts for approximately 1% [11, 12]. MC, also known as colloid adenocarcinomas, were described over 80 years ago and have an abundance of extracytoplamsic mucin that usually comprises > 50% of the total tumour mass [13]. SC carcinoma was first described in the colorectal setting by Laufman and Saphir in 1951 as ‘linitis plastica’ of the colon [14]. It classically consists of cells with peripherally placed nuclei displaced by intracytoplasmic mucin in > 50% of the total tumour mass. Signet-ring cells exist either as single cells or as loose clusters and spread diffusely throughout the bowel wall implying loss of cell–cell adhesion at the molecular level [15].

The histological subtypes of colorectal cancer are believed to have prognostic relevance. It has been suggested that SC and MC tumours are associated with advanced stage at presentation [16] and a poor prognosis [17] compared to AC. However, it is still unclear whether the prognosis of SC and MC is related to the advanced stage of the disease at presentation or whether it is related to the primary tumour characteristics. The purpose of this study, through a systematic review with meta-analyses of the available literature, is to investigate and compare local and systemic recurrence patterns and long-term survival of SC and MC adenocarcinomas compared to AC. Differences in patient and disease-specific variables such as age, gender, tumour location and staging are also analysed. This in turn should help provide further understanding and guidance on how to manage the different subgroups of colorectal cancer.

## Methods

We conducted this systematic review and meta-analysis according to the Preferred Reporting Items for Systematic reviews and Meta-Analysis group (PRISMA) guidelines [18] and the Cochrane Handbook for Systematic Reviews of Interventions [19]. The work was registered in the PROSPERO database for systematic reviews in August 2020 (CRD42020188060).

### Search strategy

A literature search of MEDLINE, EMBASE, Ovid and The Cochrane library was performed. Specific research equations were formulated for each database using the following Medical Subject Headings (MeSH) terms: *signet cell*-*ring adenocarcinoma/cancer, mucinous adenocarcinoma/cancer, mucin, colorectal adenocarcinoma/cancer, comparative study, local/locoregional recurrence, metastasis and survival*. We retrieved articles published in English from January 1, 1985, to May 30, 2020, that reported outcomes on SC and MC versus AC. The reference lists from the selected studies were reviewed to identify additional relevant studies.

### Study selection and data extraction

Studies were included if they met the following criteria: (1) studies were comparative between SC and/or MC with AC, (2) at least one outcome of recurrence and survival pattern was reported, (3) studies reported outcomes at least at yearly intervals throughout the duration of follow-up to enable hazard ratio (HR) calculations, (4) the site of the cancer had to be specified as ‘colon’ or ‘rectum’, (5) definitions of SC and MC had to be reported according to the World Health Organisation criteria or equivalent [20]. Exclusion criteria were the following: (1) studies reporting on appendiceal tumours, benign/premalignant tumours or adenomas or non-colonic primary adenocarcinomas, (2) articles published in a non-English language or in a book, (3) letters to the editor or case reports, (4) non-comparative studies to AC, (5) no reported outcomes on recurrence rates or survival.

Two authors conducted the search and identification independently, and a third author confirmed that the final selected manuscripts met the inclusion criteria. The two authors independently extracted the following information from the included studies: first author, year of publication, country, patient number, study design, age, gender, tumour location, stage (as defined by the American Joint Committee of Cancer, AJCC) [21] and tumour size (cm). The following outcomes were also recorded: locoregional recurrence rate and systemic recurrence rate according to stage, and 5-year overall survival from the time of diagnosis.

### Statistical analysis

The weighted mean difference (WMD) was used to analyse continuous variables, such as patient age and tumour size, accounting for sample size and event rate. Standard deviation (SD) was calculated using statistical algorithms. The odds ratio (OR) was used as the statistical measure for all other dichotomous outcomes. The OR represents the odds of an adverse event (e.g. recurrence) occurring in the experimental group (SC and MC) versus the control group (AC) and approximates the relative risk. An OR of greater than one indicated greater risk of an adverse event happening in the SC or MC groups.

The logarithm of the HR with 95% confidence intervals (CI) was used as the primary summary statistic as described by Parmar et al. [22]. The estimate of HR and its variance was either extracted directly from the study or required additional calculation depending on the method of data presented: annual mortality rates, survival curves, number of deaths or percentage freedom from death. Calculation of the logarithm of the HRs and their 95% CI was also performed yearly for the first three years after treatment. Meta-analysis of data was conducted using a random-effects model. Publication bias was explored graphically with funnel plots to detect asymmetry and any outliers. Inter-study heterogeneity was assessed using the Chi^2^-statistic and the *I*^2^ value to measure the degree of variation not attributable to chance alone. This was graded as low (*I*^2^ < 25%), moderate (*I* ^2^ = 25–75%) or high (*I*^2^ > 75%). This study was performed in line with Cochrane recommendations and following PRISMA guidelines and using the statistical software STATA V 12.

### Quality assessment of studies

The quality of all observational studies was assessed using the Newcastle–Ottawa Scale (NOS) [23]. This was calculated by examining three factors: method of patient selection, comparability of the study groups and number of outcomes reported. The full score was nine stars, and studies that had a score of seven stars or more were considered to be of higher quality. Two reviewers independently assessed the quality of the study and any disagreement was resolved by re-examining the relevant paper until consensus was achieved.

## Results

**Fig. 1:**
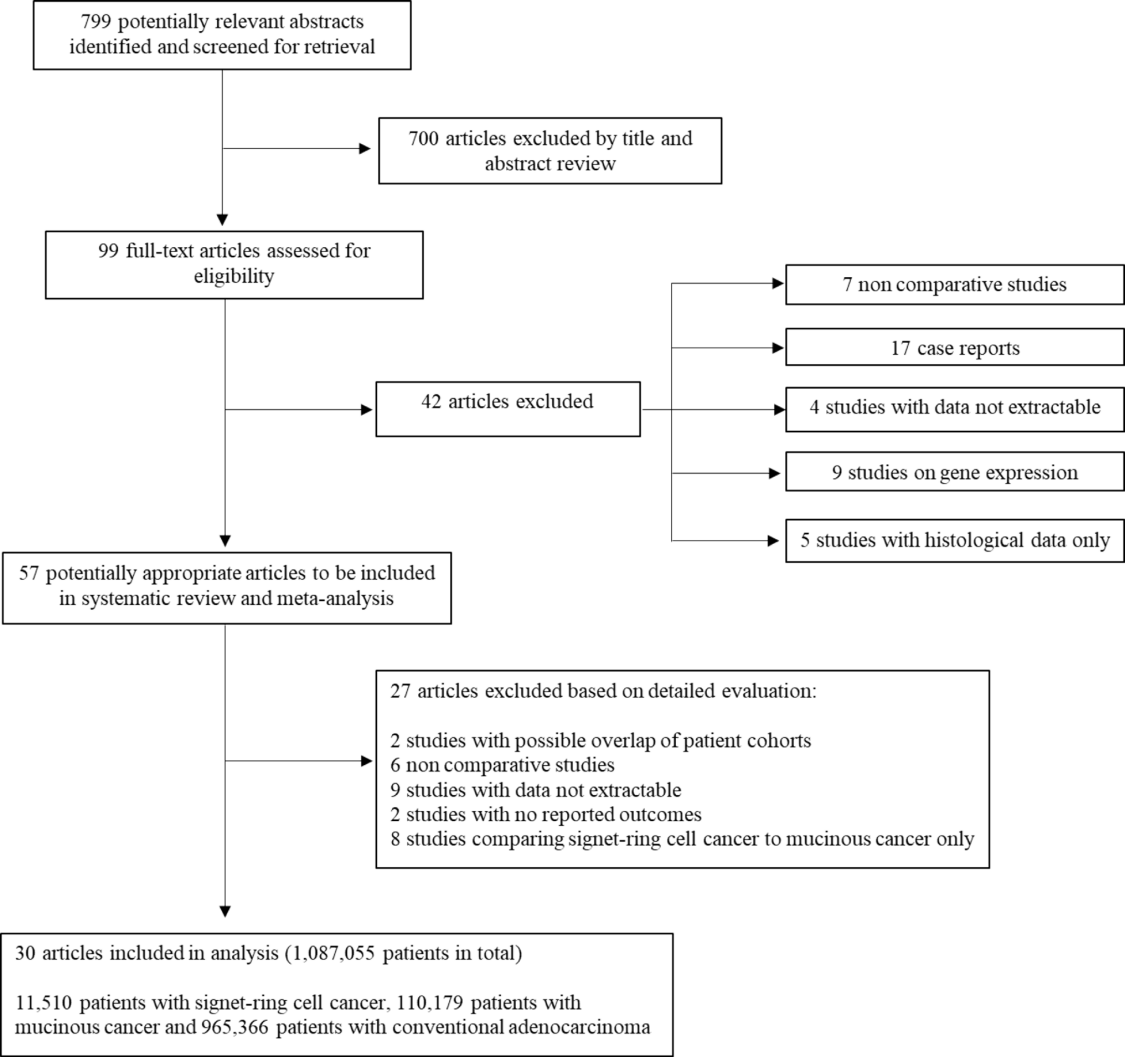
The flowchart shows the literature search and study selection process according to the PRISMA guidelines

A total of 799 published articles were identified from the initial search, of which 700 were excluded based on title and abstract review. The remaining 99 articles underwent full-text evaluation and 69 studies were further excluded. The remaining 30 comparative studies, comprising a total of 1,087,055 patients, were eligible and were included in the qualitative and quantitative analyses [10, 14, 16, 17, 24–49]. The PRISMA diagram of the literature search is shown in Fig. 1.

Fifteen studies evaluated outcomes for all three types of cancer [10, 14, 25–27, 31, 32, 34, 37, 39, 42–45, 47]. Six studies compared SC to AC only [16, 24, 35, 40, 41, 49] and nine studies compared MC to AC only [17, 28–30, 33, 36, 38, 46, 48]. The final analysis included 11,510 (1.06%) patients with SC, 110,179 (10.13%) with MC and 965,366 (88.81%) patients with AC. Nine studies were prospective non-randomised controlled studies [17, 24, 25, 27, 31, 37, 39, 40, 44] with the remaining being retrospective. Fifteen studies were considered to be of higher quality [10, 16, 17, 25, 28, 30–32, 36–38, 40, 42, 44, 47] and 20 studies were published during or after the year 2000 [10, 14, 17, 24, 25–27, 31–37, 39, 42, 44,45,47, 49]. The demographic information, tumour location, staging for patients at presentation with SC, MC or AC and the quality scoring for the 30 individual studies included in the analysis is summarised in Additional file 1: Table S1.

### Demographic analysis

**Fig. 2:**
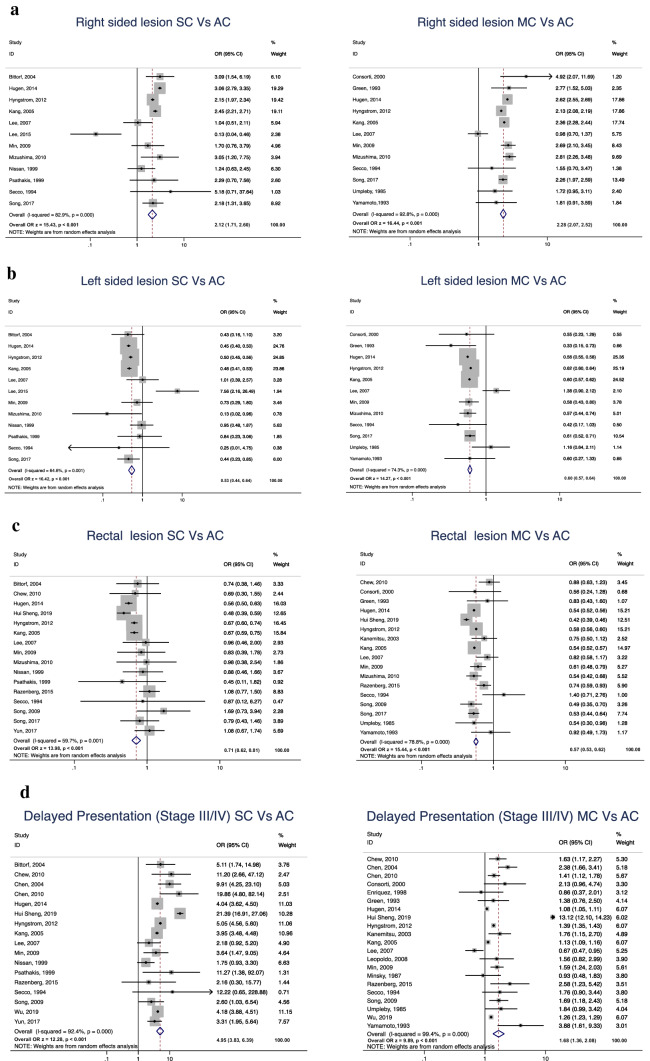
Meta-analysis and forest plot comparing **a** Right-sided lesion, **b** Left-sided lesion, **c** Rectal lesion, **d** Delayed presentation (stage III/IV) of signet-ring cell cancer and mucinous cancer to conventional adenocarcinoma. SC: signet-ring cell cancer; MC: mucinous cancer; AC: conventional adenocarcinoma; OR: odds ratio; CI: confidence interval

Age, gender, location, stage and tumour size differences between SC and MC with AC were suitable for analysis as shown in Table 1. Patients with SC and MC were found to be significantly younger than patients with AC (WMD − 0.47; 95% CI − 0.84 to − 0.10, p = 0.014 and WMD − 0.22; 95% CI − 0.44 to − 0.00; p = 0.047, respectively) (Additional file 1: Fig. S1a). No gender difference was found between SC and AC patients (OR 1.00; 95% CI 0.94–1.07; p = 0.972). There was a gender difference found between MC and AC (OR 1.14; 95% CI 1.09–1.19; p < 0.001) (Additional file 1: Fig. S1b). Both SC and MC were most commonly found in the right colon (OR 2.12; 95% CI 1.72–2.60, p < 0.001 and OR 2.28; 95% CI 2.07–2.52; p < 0.001, respectively) when compared to AC and less frequently in the left colon (OR 0.53; 95% CI 0.44–0.64; p < 0.001 and OR 0.60; 95% CI 0.57–0.64; p < 0.001, respectively) and rectum (OR 0.71; 95% CI 0.62–0.81; p < 0.001 and OR 0.57; 95% CI 0.53–0.62; p < 0.001, respectively) (Fig. 2a–c).

**Table 1:** Comparison of clinicopathological factors, recurrence and survival outcomes of signet-ring cell cancer and mucinous cancer versus conventional adenocarcinoma

	Number of studies	Total number of patients included in analysis			HR/OR/WMD	95% CI	p-value	Study heterogeneity
		SC	MC	AC				I^2^ (%)/X^2^
Demographic characteristics								
Mean age (in years)	6	1643	–	152,869	− 0.47	− 0.84 to − 0.10	0.014*	88.6/43.73
Female gender	17	11,438	–	956,150	1.00	0.94–1.07	0.972	34.2/24.33
Tumour location in right colon	12	6004	–	57,1493	2.12	1.72–2.60	< 0.001*	82.9/64.26
Tumour location in left colon	12	6004	–	569,656	0.53	0.44–0.64	< 0.001*	64.6/31.08
Tumour location in rectosigmoid/rectum	16	7240	–	605,375	0.71	0.62–0.81	< 0.001*	59.7/37.20
Advanced stage at presentation (III/IV)	17	11,405	–	934,780	4.95	3.83–6.39	< 0.001*	92.4/211.05
Tumour diameter at presentation (cm)	5	131	–	14,527	0.60	0.24–0.96	0.001*	74.9/15.92
Local recurrence rate (at 5 years)								
Stages I–III	3	61	–	7090	2.81	1.40–5.65	0.004*	0.0/1.93
Systemic recurrence rate (at 5 years)								
Stages I–III	5	110	–	11,623	4.25	2.16–8.34	< 0.001*	49.7/7.96
Survival rate (at 5 years)								
Overall survival	18	11,458	–	955,961	2.54	1.98–3.27	< 0.001*	99.1/1971.81
Demographic characteristics								
Mean age (in years)	7	–	17,974	160,088	− 0.22	− 0.44 to − 0.00	0.047*	97.6/246.82
Female gender	22	–	110,067	944,326	1.14	1.09–1.19	< 0.001*	79.1/100.43
Tumour location in right colon	12	–	71,487	563,374	2.28	2.07–2.52	< 0.001*	92.8/152.67
Tumour location in left colon	12	–	71,487	563,374	0.60	0.57–0.64	< 0.001*	74.3/42.80
Tumour location in rectosigmoid/rectum	17	–	78,542	588,795	0.57	0.53–0.62	< 0.001*	78.8/75.64
Advanced stage at presentation (III/IV)	21	–	108,810	918,843	1.68	1.36–2.08	< 0.001*	99.4/3472.13
Tumour diameter at presentation (cm)	4	–	116	14,452	0.53	0.41–0.65	< 0.001*	71.0/10.36
Local recurrence rate (at 5 years)								
Stages I–III	10	–	1087	10,852	1.92	1.18–3.15	0.009*	74.0/34.59
Systemic recurrence rate (at 5 years)								
Stages I–III	10	–	847	11,176	1.81	1.37–2.40	< 0.001*	21.2/57.6
Survival rate (at 5 years)								
Overall survival	22	–	93,128	798,556	1.38	1.19–1.61	< 0.001*	98.6/1510.98

Table summarises data from studies that reported age, gender, tumour location, advanced stage at presentation (as defined by the American Joint Committee of Cancer), tumour diameter, 5-year recurrence (local and systemic) and five-year overall survival rates

SC: signet-ring cell cancer; MC: mucinous cancer; AC: conventional adenocarcinoma; HR: hazard ratio; OR: odds ratio; WMD: weighted mean difference, CI: confidence interval

*Statistically significant

Stage at presentation was significantly more advanced in SC (OR 4.95; 95% CI 3.83–6.39; p < 0.001) and MC (OR 1.68; 95% CI 1.36–2.08; p < 0.001) than AC (Fig. 2d). Similarly, tumour size at presentation was significantly greater for SC and MC (WMD 0.60; 95% CI 0.24–0.96; p = 0.001 and WMD 0.53, 95% CI 0.41–0.65; p < 0.001, respectively) when compared to AC (Additional file 1: Fig. S1c).

### Local and systemic recurrence analysis

**Fig. 3:**
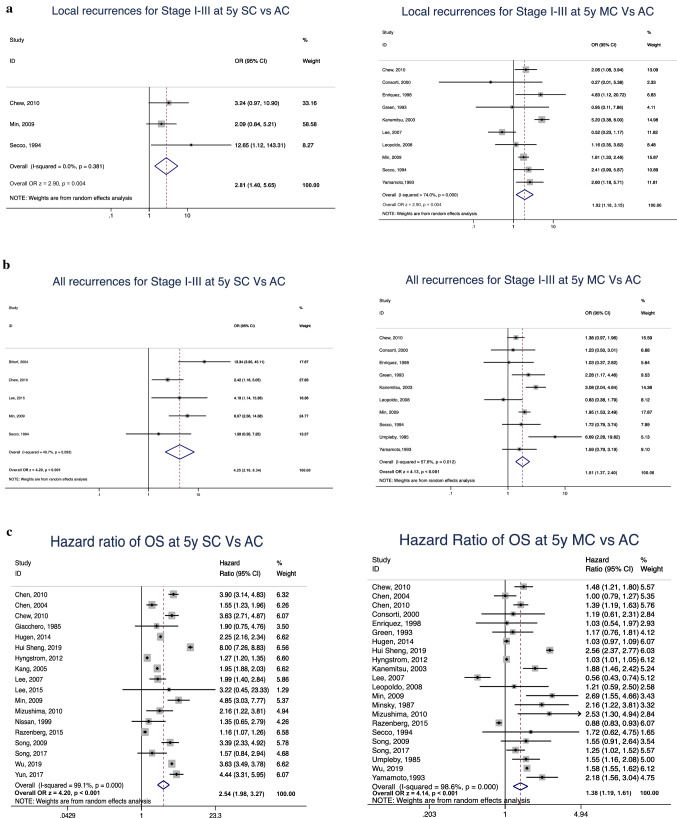
Meta-analysis and forest plot comparing **a** 5-year local recurrence for stage I–III, **b** 5-year all recurrences (local and systemic) for stage I–III, **c** 5-year overall survival of signet-ring cell cancer and mucinous cancer to conventional adenocarcinoma. SC: signet-ring cell cancer; MC: mucinous cancer; AC: conventional adenocarcinoma; OS: overall survival; OR: odds ratio; CI: confidence interval

Risk of 5-year local recurrence for stage I-III disease was higher for both SC and MC versus AC (OR 2.81; 95% CI 1.40–5.65; p = 0.004 and OR 1.92; 95% CI 1.18–3.15; p = 0.009, respectively) (Fig. 3a). Only one study included local recurrence for only stage III disease, and therefore this was not assessed in the meta-analysis. The 5-year systemic recurrence rate for stage I–III disease for SC and MC was also significantly higher when compared to AC (OR 4.25; 95% CI 1.98–3.27; p < 0.001 and OR 1.81; 95% CI; p < 0.001, respectively) (Fig. 3b).

### Survival analysis

Five-year overall survival was significantly poorer for both SC and MC when compared to AC (HR 2.54; 95% CI 1.98–3.27, p < 0.001 and HR 1.38; 95% CI 1.19–1.61; p < 0.001, respectively) (Fig. 3c). In particular, significant heterogeneity was observed when comparing the staging at presentation of SC and MC to AC (*I*^2^ 92.4% and 99.4%, respectively). Survival analysis was also subject to great between-study heterogeneity in the case of both SC and MC (*I*^2^ 99.1% and 98.6%, respectively). Begg’s and Egger’s tests were performed for all outcomes with significant heterogeneity and funnel plots were inspected without significant evidence of publication bias.

## Discussion

To our knowledge this study provides the first systematic review and meta-analysis of the clinicopathological characteristics and survival outcomes for SC and MC compared to AC. Thirty studies with a combined population of 1,087,055 patients met the inclusion criteria and were included in the final meta-analysis. The results highlight the important clinical implications associated with the different histological subgroups of colorectal cancer. We have identified the behaviour of SC and MC and their prognostic significance which should provide guidance on how to further manage SC and MC of the colon and the rectum. Key factors to improve survival include closer follow-up, earlier detection of recurrent disease, efficacy of systemic therapies, resection of metastases and implementation of a ‘continuum of care’ [50, 51].

Our meta-analysis has shown that SC and MC present at an earlier age, by 0.47 and 0.22 years respectively, compared to AC. The histological subtypes of SC and MC may be contributing to the increase in incidence seen in colorectal cancer in younger adults. There is also significantly more chance of SC and MC developing in the right colon, presenting at an advanced stage. Local recurrence at five years for SC and MC were more likely than AC, with a poorer overall survival at five years.

Accumulation of mutations in various oncogenes and tumour suppressor genes drives the development of colorectal cancer [52]. Results from the present study may be explained to a certain extent by considering the molecular genetics of SC and MC and the differences from AC. E-cadherin is a calcium-dependent cell-to-cell adhesion molecule that has been implicated in the biological behaviour of SC. It has been suggested that e-cadherin together with catenin can act as an ‘intercellular glue’ with a suppressor role in tumour invasion in colorectal cancer [53]. Downregulation of e-cadherin in association with locally infiltrative behaviour and peritoneal metastases has been shown in gastric SC and was associated with poorer prognosis [54, 55]. In a study by Kim et al. [56] in 2002, 100% of colorectal SC was shown to have markedly reduced or absent expression of e-cadherin when compared to only 23.5% of AC (p < 0.05). Peritoneal metastases and a locally aggressive behaviour in colorectal cancer was found in association with e-cadherin proteolysis [57]. Another molecular complex known as ‘trefoil factor’ has a major role to play in mucosal healing by disturbing the complexes among e-cadherin and β-catenin and allowing epithelial proliferation [58]. ‘Trefoil factor’ and mucin are often co-expressed leading to the theory that overexpression of mucin in SC and MC leads to a secondary overexpression of ‘trefoil factor’ and disruption of the E-cadherin/β-catenin complexes [59]. It has also been suggested that due to the mucin content of SC and MC, local immune host recognition as tumour cells is evaded allowing easier locoregional spread [60]. Loss of e-cadherin may be key in disruption of cell-to-cell cohesion, allowing a more locally invasive and infiltrating behaviour involving the surrounding structures, peritoneum and lymph nodes. This may in turn explain the more advanced stage at presentation of these cancers as well as the metastatic pattern and aggressive behaviour.

Other studies have also made similar observations regarding tumour location of MC and SC. Benedix et al. [61] in a study of 17,641 patients found that right-sided cancers were more often poorly differentiated or of SC/MC morphology and presented more commonly with peritoneal metastases compared to left-sided tumours that presented with liver metastatic disease. This observation was enhanced in a review by the same author reaching similar conclusions [62] and indeed suggesting that tumours at different colonic locations may be different entities. However, the study from Lee et al. [14] that included poorly differentiated AC showed equal preponderance of AC to MC in the right colon.

Our study seems to indirectly suggest a continuum of poorer prognosis that may not only depend on tumour type but on tumour location as well. Analysis of survival based on tumour type as well as location was not possible from the included studies although other authors have suggested location to be an independent predictor or survival [63–69]. Chew et al. [25] on the other hand using a multivariate Cox proportional hazards model showed independent association of histological subtype but not tumour location to survival. These findings are consistent with the results of the present meta-analysis where histological subtype is the direct determinant of the colonic region involved with tumour type dictating survival and locoregional recurrence patterns. SC seems to have an inherently more aggressive biological behaviour even to poorly differentiated AC, and results in poorer survival outcomes. Local recurrence appears to be frequent in both SC and MC. This may be directly related to the above-mentioned hypothesis of e-cadherin loss that can occur at the onset of carcinogenesis resulting in a more aggressive locoregional behaviour [55].

### Limitations

This study is subject to some limitations that must be addressed. Between-study heterogeneity was evident particularly in staging at presentation and survival outcomes. Attempts were made by the authors to statistically account for this with a random-effects model analysis to allow for differences between studies and treatment centres. There was no significant publication bias found within the statistical powers of the Begg’s and Egger tests. Heterogeneity has been reduced by employing these techniques but not abolished as there are factors inherent to the studies that cannot be controlled by meta-analytical techniques. A different mix of populations from the included studies may account for the heterogeneity observed in the demographic characteristics.

Multimodal treatments, surgical approaches and techniques as well as imaging tools and histopathological classifications have changed significantly over the last few decades which makes the comparison of the results more difficult. Tumour grade was another important factor that could not be accounted for as only one study was matched for tumour grade [41], one study included only poorly differentiated AC [14] while another included only well and moderately differentiated AC [39]. Studies were non-randomised as is frequently the case in surgical research but meta-analysis of non-randomised studies has been shown to be a useful tool in its own remit. Moreover, the overall level of evidence emerging from the literature were judged as high in the majority of cases.

## Conclusions

Based on the available evidence, SC and MC present and behave in a pattern distinct from AC. SC seems to be an aggressive type of colorectal cancer presenting in younger patients, in the right colon and at a more advanced stage. This in turn leads to a poorer stage-by-stage survival and a higher probability of local recurrence rates when compared to AC. MC seems to behave in a similar fashion to SC in terms of local recurrence and overall survival. These factors need to be taken into consideration when planning surgical and oncological management of such cancers. Follow-up may be needed more regularly for SC and MC compared to AC due to the higher recurrence rates. Local control would also be important in the case of SC and MC with implications on the need for careful lymphadenectomy and/or peritonectomy with or without neoadjuvant therapy. Post-resection surveillance protocols may need to be adjusted to detect local and peritoneal spread early and this could form the basis of future trials.

## Supplementary Information

Below is the link to the electronic supplementary material.

Additional file 1: **Table S1.** Demographic details, tumour location and staging and quality scoring for the thirty studies included in the analysis. SC = signet-ring cell cancer, MC = mucinous cancer, AC = conventional adenocarcinoma; - = not available/not extractable, ‡ = includes mucinous adenocarcinoma, ⁞ = includes poorly differentiated conventional adenocarcinoma, ▲ = proximal colon/distal colon, • = colon/rectum; R = retrospective, PNR = prospective non-randomised; RC = right colon; TC = transverse colon; LC = left colon; R = rectum; AJCC = American Joint Committee on Cancer; NOS = Newcastle–Ottawa score (maximum score of nine). **Figure S1.** Meta-analysis and forest plot comparing (a) Age, (b) Gender, (c) Tumour diameter (cm) of signet-ring cell cancer and mucinous cancer to conventional adenocarcinoma. SC = signet-ring cell cancer, MC = mucinous cancer, AC = conventional adenocarcinoma, OR = odds ratio, CI = confidence interval.

## Data Availability

The datasets used generated during and/or analysed during the current study are available from the corresponding author on request.
