# Supplementary material for: Clinicopathological factors and survival outcomes of signet-ring cell and mucinous carcinoma versus adenocarcinoma of the colon and rectum: a systematic review and meta-analysis
Source: Discov Oncol. 2021 Feb 22;12:5. doi: 10.1007/s12672-021-00398-6 (PMC8762524; doi:10.1007/s12672-021-00398-6)
Supplement: Supplementary file 1 — Additional file 1: Table S1. Demographic details, tumour location and staging and quality scoring for the thirty studies included in the analysis. SC = signet-ring cell cancer, MC = mucinous cancer, AC = conventional adenocarcinoma; - = not available/not extractable, ‡ = includes mucinous adenocarcinoma, ⁞ = includes poorly differentiated conventional adenocarcinoma, ▲ = proximal colon/distal colon, • = colon/rectum; R = retrospective, PNR = prospective non-randomised; RC = right colon; TC = transverse colon; LC = left colon; R = rectum; AJCC = American Joint Committee on Cancer; NOS = Newcastle–Ottawa score (maximum score of nine). Figure S1. Meta-analysis and forest plot comparing (a) Age, (b) Gender, (c) Tumour diameter (cm) of signet-ring cell cancer and mucinous cancer to conventional adenocarcinoma. SC = signet-ring cell cancer, MC = mucinous cancer, AC = conventional adenocarcinoma, OR = odds ratio, CI = confidence interval. [file 12672_2021_398_MOESM1_ESM.docx]

**Additional file**

| **Authors et al. (year)** | **Type** |  | **Patients** |  |  | **Mean age (years)** | | | **Female gender (%)** | | | **Tumour location (RC/TC/LC/R) (%)** | | | **AJCC stage III/IV (%)** | | | **Quality (NOS)** |
| --- | --- | --- | --- | --- | --- | --- | --- | --- | --- | --- | --- | --- | --- | --- | --- | --- | --- | --- |
|  |  | **SC** | **MC** | **AC** | **SC** |  | **MC** | **AC** | **SC** | **MC** | **AC** | **SC** | **MC** | **AC** | **SC** | **MC** | **AC** |  |
| Bittorf et al. [24], 2004 | PNR | 34 | - | 4458‡ | 57 |  | - | 63 | 56 | - | 58 | 29/9/15/47 | - | 13/4/29/54 | 59 | - | 47 | 6* |
| Chew et al. [25], 2010 | PNR | 30 | 167 | 2567 | 64 |  | 62 | 66 | 73 | 53 | 55 | 27/-/50/23 | 30/-/38/32 | 19/-/46/35 | 94 | 67 | 56 | 8* |
| Chen et al. [26], 2004 | R | 61 | 144 | 2414 | 48 |  | 57 | 63 | 33 | 47 | 41 | -/-/-/46 | -/-/-/34 | -/-/-/29 | 90 | 69 | 48 | 5* |
| Chen et al. [27], 2010 | PNR | 45 | 332 | 2984 | 54 |  | 60 | 62 | 40 | 52 | 48 | 62/38^▲^ | 61/39^▲^ | 38/62^▲^ | 52 | 6 | - | 5* |
| Connelly et al. [28], 1991 | R | - | 60 | 120 | - |  | 63 | - | - | 58 | - | - | 39/-/18/43 | - | - | 44 | - | 8* |
| Consorti et al. [17], 2000 | PNR | - | 29 | 212 | - |  | 61 | 65 | - | 48 | 47 | - | 38/-/28/31 | 12/-/43/44 | - | 62 | 43 | 7* |
| Enriquez et al. [29], 1998 | R | - | 27 | 115 | - |  | 60 | 67 | - | 40 | 48 | - | 48/52^▲^ | 24/76^▲^ | - | 55 | 59 | 6* |
| Giacchero et al. [16], 1985 | R | 9 | - | 45 | 52 |  | - | - | 78 | - | - | 22/-/22/56 | - | - | 100 | - | - | 7* |
| Green et al. [30], 1993 | R | - | 52 | 345 | - |  | 67 | 66 | - | - | - | - | 34/24/14/12 | 20/12/36/32 | - | 50 | 41 | 7* |
| Hugen et al. [31], 2014 | PNR | 1972 | 26851 | 167934 | 70 |  | 72 | 71 | 70 | 72 | 71 | 58/-/22/17 | 55/-/26/17 | 32/-/38/28 | 75 | 48 | 44 | 7* |
| Hui Sheng et al. [10], 2019 | R | 814 | 5689 | 15812 | - |  | - | - | 49 | 52 | 46 | 88/12• | 89/11• | 77/23• | 69 | 44 | 19 | 7* |
| Hyngstrom et al. [32], 2012 | R | 2260 | 25546 | 216988 | - |  | - | - | 50 | 54 | 51 | 62/-/19/20 | 60/-/22/18 | 42/-/31/27 | 80 | 52 | 44 | 8* |
| Kanemitsu et al. [33], 2003 | R | - | 97 | 2197 | - |  | 55 | 59 | - | 47 | 39 | - | -/-/-/46 | -/-/-/54 | - | 60 | 46 | 6* |
| Kang et al. [34], 2005 | R | 1522 | 16991 | 146115 | 66 |  | 70 | 70 | 49 | 53 | 49 | 50/10/19/21 | 50/9/23/18 | 32/7/32/29 | 81 | 53 | 49 | 5* |
| Lee et al. [14], 2007 | R | 35 | 294 | 252 | 48 |  | 53 | 54 | 37 | 58 | 46 | 46/-/17/37 | 44/-/22/34 | 45/-/17/38 | 80 | 55 | 65 | 5* |
| Lee et al. [35], 2015 | R | 15 | - | 75 | 56 |  | - | 56 | - | - | - | 27/-/73/- | - | 73/-/27/- | 80 | - | 80 | 6* |
| Leopoldo et al. [36], 2008 | R | - | 60 | 96 | - |  | 70 | 69 | - | 52 | 47 | - | -/-/-/13 | -/-/-/13 | - | 52 | 41 | 7* |
| Min et al. [37], 2009 | PNR | 27 | 278 | 4214 | 47 |  | 54 | 59 | 44 | 36 | 40 | 33/-/22/45 | 44/-/19/37 | 23/-/28/49 | 78 | 60 | 49 | 8* |
| Minsky et al. [38], 1987 | R | - | 49 | 376 | - |  | 69 | 67 | - | 55 | 45 | - | - | - | - | 27 | 28 | 8* |
| Mizushima et al. [39], 2010 | PNR | 19 | 375⁞ | 5417 | 66 |  | 66 | 66 | 58 | 48 | 42 | 37/16/5/37 | 39/13/20/25 | 19/9/31/39 | 84 | 52 | 42 | 6* |
| Nissan et al. [40], 1999 | PNR | 40 | - | 3371 | 61 |  | - | 64 | 52 | - | 83 | 20/10/30/40 | - | 18/8/31/43 | 60 | - | 45 | 8* |
| Psathakis et al. [41], 1999 | R | 14 | - | 56 | 68 |  | - | - | - | - | - | 43/7/29/21 | - | 25/2/36/38 | 93 | - | 50 | 5* |
| Razenberg et al. [42], 2015 | R | 311 | 958 | 3008 | - |  | - | - | 44 | 50 | 49 | 85/15• | 89/11• | 86/14• | 92 | 85 | 87 | 7* |
| Secco et al. [43], 1994 | R | 4 | 39 | 309 | 60 |  | 65 | 62 | 100 | 39 | 50 | 50/-/-/50 | 10/13/15/62 | 9/7/30/54 | 100 | 56 | 42 | 6* |
| Song et al. [44], 2009 | PNR | 25 | 144 | 1837 | 40 |  | 52 | 59 | 64 | 37 | 42 | -/-/-/68 | -/-/-/38 | -/-/-/56 | 80 | 59 | 52 | 7* |
| Song et al. [45], 2017 | R | 62 | 934 | 20467 | - |  | - | - | 47 | 51 | 47 | - | - | - | 78 | 52 | 45 | 6* |
| Umpleby et al. [46], 1985 | R | - | 54 | 576 | - |  | - | - | - | 54 | 50 | - | 19/17/33/31 | 24/30/46 | - | 72 | 58 | 6* |
| Wu et al. [47], 2019 | R | 4140 | 30965 | 349891 | - |  | - | - | 47 | 51 | 47 | - | - | - | 78 | 52 | 45 | 8* |
| Yamamoto et al. [48], 1993 | R | - | 44 | 545 | - |  | 63 | 60 | - | 52 | 43 | - | 27/2/19/52 | 14/5/28/53 | - | 86 | 62 | 5* |
| Yun et al. [49], 2017 | R | 71 | - | 12570 | - |  | - | - | 35 | - | 40 | 59/41• | - | 61/29• | 69 | - | 43 | 6* |
| **Total** |  | **11510** | **110179** | **965366** |  |  |  |  |  |  |  |  |  |  |  |  |  |  |

**Table S1.** Demographic details, tumour location and staging and quality scoring for the thirty studies included in the analysis. SC = signet-ring cell cancer, MC = mucinous cancer, AC = conventional adenocarcinoma; - = not available/not extractable, ‡ = includes mucinous adenocarcinoma, ⁞ = includes poorly differentiated conventional adenocarcinoma, ▲=proximal colon/distal colon, • = colon/rectum; R = retrospective, PNR = prospective non-randomised; RC = right colon; TC = transverse colon; LC = left colon; R = rectum; AJCC = American Joint Committee on Cancer; NOS = Newcastle-Ottawa score (maximum score of nine).

**-0.22 (-0.44, -0.00) 100.00 100.00**

**Overall OR z = 1.99, p = 0.047**

**-0.47 (-0.84, -0.10) 100.00 100.00**

**Overall OR z = 2.46, p = 0.014**

a

**1.00 (0.94, 1.07) 100.00**

**1.14 (1.09, 1.19) 100.00 100.00**

**Overall OR z = 5.35, p < 0.001**

**Overall OR z = 0.04, p = 0.972**

b

**0.53 (0.41, 0.65) 100.00**

**Overall OR z = 8.95, p < 0.001**

**0.53 (0.44, 0.64) 100.00**

**Overall OR z = 3.29, p = 0.001**

c

**Figure S1.** Meta-analysis and forest plot comparing (a) Age, (b) Gender, (c) Tumour diameter (cm) of signet-ring cell cancer and mucinous cancer to conventional adenocarcinoma. SC = signet-ring cell cancer, MC = mucinous cancer, AC = conventional adenocarcinoma, OR = odds ratio, CI = confidence interval.
